# Advances in Transgenic Mouse Models to Study Infections by Human Pathogenic Viruses

**DOI:** 10.3390/ijms21239289

**Published:** 2020-12-05

**Authors:** Dörthe Masemann, Stephan Ludwig, Yvonne Boergeling

**Affiliations:** Institute of Virology Muenster, University of Muenster, 48149 Muenster, Germany; d.masemann@uni-muenster.de (D.M.); ludwigs@uni-muenster.de (S.L.)

**Keywords:** human pathogenic viruses, transgenic mice, humanized mouse models

## Abstract

Medical research is changing into direction of precision therapy, thus, sophisticated preclinical models are urgently needed. In human pathogenic virus research, the major technical hurdle is not only to translate discoveries from animals to treatments of humans, but also to overcome the problem of interspecies differences with regard to productive infections and comparable disease development. Transgenic mice provide a basis for research of disease pathogenesis after infection with human-specific viruses. Today, humanized mice can be found at the very heart of this forefront of medical research allowing for recapitulation of disease pathogenesis and drug mechanisms in humans. This review discusses progress in the development and use of transgenic mice for the study of virus-induced human diseases towards identification of new drug innovations to treat and control human pathogenic infectious diseases.

## 1. Introduction

In times of a global pandemic, it is more obvious than ever that human pathogenic viruses are a major threat to global health. The emergence of human pathogenic viruses such as SARS-CoV-2 (severe acute respiratory syndrome coronavirus 2) or Zika virus highlights the urgent need of representative preclinical models to study virus entry, replication and spread within an organism as tools to develop efficient antiviral therapies and vaccinations. Aside from that, also several well-known human pathogenic viruses that are characterized in molecular detail like influenza virus, human immunodeficiency virus (HIV) or hepatitis viruses still remain a huge threat to public health.

**Figure 1:**
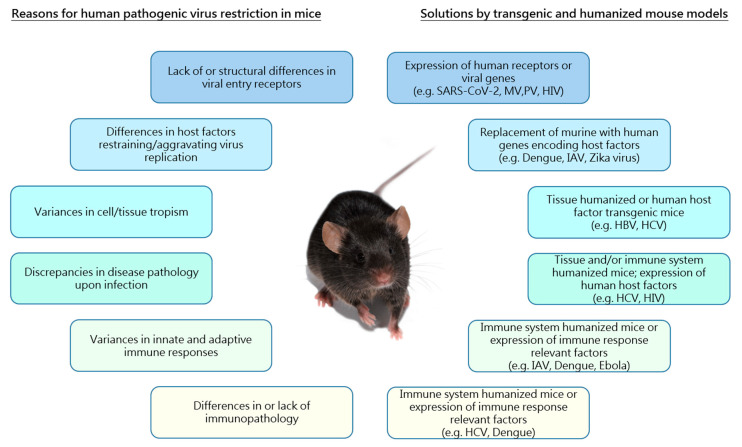
Reasons underlying restricted species specificity of human pathogenic viruses and solutions provided by the use of transgenic and humanized mouse models. Schematic overview of limitations in the use of mouse models for research of human pathogenic viruses (left) and examples of different transgenic/humanized mouse models providing solutions to overcome the respective restrictions (right). Associated boxes (limitations/solutions) are indicated by the use of the same color. MV: Measles virus; PV: Poliovirus; HIV: Human immunodeficiency virus; IAV: Influenza A virus; HBV: Hepatitis B virus; HCV: Hepatitis C virus.

Mechanisms of infection, replication and disease pathology differ widely among different viruses. Suitability of cell culture systems or organoids to obtain comprehensive knowledge about viral characteristics or to test antiviral approaches is highly limited due to the lack of a functional immune system and vasculature. Thus, animal models still represent a unique opportunity to study viral replication processes and antiviral agents in vivo. Rodent models, and especially mice, are widely accepted and the preferred preclinical systems as they are readily available and exhibit low maintenance costs. However, viruses coevolve with their host organisms and, thus, often show a restricted species specificity frequently complicating the research of human pathogenic viruses in mouse models. This narrow host range is often based on the lack of surface entry receptors necessary for virus infection or due to remarkable differences in murine and human innate immune responses upon viral infection. Accompanying this, tissue tropism of human pathogenic viruses can differ significantly in mice causing differences in virus-induced pathology (Figure 1). Hence, mouse models often demonstrate limited susceptibility or permissiveness and, thus, results obtained from studies in murine models cannot necessarily be extrapolated to humans, which particularly complicates the validity of drug development and vaccination studies.

Genetically modified and transgenic mouse models represent a sophisticated possibility to overcome interspecies differences in human pathogenic virus research such as species specificity and tissue tropism, allowing to investigate the function of specific human entry receptors, host factors or innate and adaptive immune responses in immunocompetent organisms. These models were of significant importance for scientific progress within the last decades and provided immeasurable insights into virus biology and disease pathogenesis. A decisive milestone was the development of immune system or tissue-humanized mouse models facilitating infection and replication of human pathogenic viruses in a humanized system. With recent advances in human cell or tissue engraftment in transgenic humanized mice, antiviral strategies and vaccines against many human pathogenic viruses already have been or will be evaluated in the future, towards identification of new drug innovations to treat and control human pathogenic infectious diseases. 

This review aims to provide an overview of different options to genetically engineer and humanize mice. We will discuss several examples of human pathogenic viruses that have been studied in translational, human-relevant preclinical systems (see Table 1).

## 2. Transgenic Mice Expressing Viral Genes

Due to the absence of permissive mouse models for several human viruses, transgenic mouse strains were developed incorporating essential individual viral genes or even whole virus genomes within their own genome to investigate virus pathogenicity and characterize specific properties of viral genes.

Some of the most intensively studied transgenic mouse models expressing viral genomes have been generated to analyze hepatitis B and C virus (HBV, HCV)-induced liver pathology and virus replication. Hepatitis B and C infection share a common phenotype of liver pathology induced by chronic necroinflammation resulting in liver cirrhosis and hepatocellular carcinoma (HCC). HBV cccDNA (covalently closed circular DNA) persists in infected host cells and the virus can uniquely integrate its genome into the DNA of the infected cell. Most integration events retain the HBx antigen [1]. In sharp contrast, HCV is a positive-strand RNA virus that replicates through a negative-stranded intermediate in the cytoplasm. Persistence of HCV is thus facilitated by RNA intermediates that remain in infected host cells. Since murine hepatocytes do not express HBV- and HCV-specific receptors, intrahepatic infection and virus spread cannot be analyzed in wild type mice [2,3].

In order to study the role of individual HBV genes in the development of hepatopathology, immunotolerant HBV-transgenic mice were generated by germline modifications. While the expression of the viral envelope protein did not induce hepatocellular cytopathology, adoptive transfer of virus–antigen primed splenocytes resulted in hepatocellular injury and immunopathology caused by MHC I-restricted antigen-presentation to CD8+ T lymphocytes and envelope-specific antibodies [4]. These data provided the first hints for virus-induced immunopathology based on adaptive immune responses directed against hepatocytes expressing viral genes. Many transgenic lineages that express individual HBV genes such as the *envelope* [5,6,7], *core* [8], *precore* [9] and *X* [10,11] genes under the control of HBV- or murine liver-specific promoters have been described in the last decades. This led to substantial improvement in understanding the properties of the respective viral genes. Afterwards, transgenic mouse models were generated expressing the full viral replicon, which resulted in high-yield HBV replication in murine kidneys and liver [12]. Using these HBV transgenic mice, efficacy of antiviral agents such as HBV-specific inhibitors, small interfering RNAs (siRNAs), and cytokines have been tested [13,14,15,16]. Other transgenic mice, induced by microinjection of viral persistence-inducing replication intermediate cccDNA [17,18] or by adeno-associated virus in vivo transduction of the HBV replicon, were used to study antiviral agents or vaccination approaches for acute or chronic infections [19,20]. More recent approaches used HBV transgenic mice to investigate the therapeutic potential of CRISPR/Cas9 techniques for use in chronic HBV gene therapy [21,22]. 

Several groups have generated transgenic mice expressing individual HCV proteins or viral polyproteins to study the effect on liver pathology, steatosis and HCC manifestations [23,24,25,26,27,28,29]. However, since the overexpression of HCV proteins in these transgenic mice differs compared to low level expression during natural infection, it is unclear whether the obtained findings are transferable to the pathology observed in humans [30]. Along that line, it was shown that inflammation-associated hepatocarcinogenesis depends on the host genetic background of the respective transgenic mouse model [31]. Thus, these models helped to elucidate the pathophysiology of HCV gene products, but they are limited by their inability to support HCV replication based on the lack of negative-stranded RNA expression. Compared to transgenic mouse models for HBV, expression of the HCV genome did not result in viral progeny production or virus genome replication. Thus, these models do not allow investigation of antiviral agents or vaccination approaches.

Since murine hepatocytes do not express the respective virus entry receptors for HBV and HCV, the abovementioned studies did neither allow for virus infection studies nor resemble the whole infection process via the natural route and virus spread as observed within human tissues [16]. Though limited in their applicability for virus infection studies, these models were groundbreaking for initial hepatotropic virus research related to the mechanisms of HBV and HCV pathogenesis. 

In line with HBV and HCVtg mice, several attempts have been made to generate HIVtg mice resulting in substantial understanding of virus–host interactions and clinical manifestation of acquired immune deficiency syndrome (AIDS) symptoms or the development of Kaposi’s sarcoma [32,33,34,35,36]. However, HIVtg mice showed discrepancies in tissue- and immunopathology (reviewed in [37]). Along that line, mice do neither express respective entry receptors nor the co-factors needed for efficient viral replication, and, thus, do not allow analyses of virus replication, antiviral strategies or vaccination approaches [38]. 

Further examples of transgenic mouse models expressing HBV, HCV or HIV transgenes can be found in Table 1.

**Table 1:** Overview of transgenic mouse models used to study infections by a selection of human pathogenic viruses. This table can only give an overview and makes no claim to completeness.

Virus	Entry Receptors	Virus Genome Transgenic Mice	Entry Receptor Transgenic Mice	Transgenic Mice with Changes in Host Factor Expression	Transgenic Tissue/Immune System Humanized Mice
HIV	CD4, CCR5, CXCR4	vHIV^wt^tg [32,33,34] vHIV*^tat^*tg [35,36,37,38,39,40,41] HIV*^nef^*tg [42,43,44,45,46,47,48,49] HIV*^delgag^*^/*pol*^tg [50] HIV*^delgag^*^/*pol*/*env*^tg [51]	*hCD4/hCCR5* [52,53]	*hCD4/hCCR5/ hcyclin T1* [54] *CCR5^-/-^*/HIV*^gp120^*tg [55]	MISTRG [56] DRAG [57] NOG-EXL [58]
Polio	PVR/CD155		*hPVR* [59,60,61,62,63,64]	*hPVR*/*IFNAR^−/−^* [65]	
Measles	CD46, CD150, Nectin-4		*hCD46* [66,67] *hCD150* [68,69] *hCD46/hCD150* [70]	*hCD46/IFNAR^−/−^* [71,72]	
HCV	CD81, Occludin, CLDN1; CARB1	vHCV*^NS3^*^/*4A*^tg [23] vHCV*^core^*^,*E1*,*E2*^tg [24] vHCV*^E1^*^,*E2*^tg [25] vHCV*^FL^*^-*N*^tg, vHCV*^S^*^-*N*^tg [27] vHCV*^core^*tg [29]	*hCD81/hOccludin* [73,74,75]		*AFC8*-hu-HSC/Hep [76,77] *MUP-uPA* [78]
HBV	NTCP	vHBVtg [6,12,79,80] vHBV*^envelope^*tg [4] vHBV*^delcore^*tg [5] vHBV*^core^*tg [8] vHBV*^HBeAg^*tg [9] vHBV*^HBsAg^*^,*pre-S*,*X*^tg [7] vHBV*^HBx^*tg [10,11] vHBV*^HBs^*tg [81] vHBV*^preS^*^/*S*^tg [82]		vHBVtg/*HNF1^−/−^* [83] vHBV*^HBs^*tg/*Abcb4^−/−^* [84]	A2/NSG—hu HSC/Hep [85] *MUP-uPA* [78,86,87]
SARS-CoV-2	ACE-2, TMPRSS2		*hACE2* [88,89,90,91,92]		
Influenza	S,3-/2,6-sialic acid residues			*hMxA* [93] *hHLA-A2* [94,95] *hHLA-DR3* [96]	DRAGA [97]
Dengue	Heparan sulfate, DC-SIGN/L-SIGN, mannose receptor, laminin receptor			*hTNFα* [98] *hHLA-A, B* [99] *IFNAR^−/−^/HLA-A, B, DRB1* [100,101]	NSG-A2 [102] NSG-SGM3 [103]
Ebola	Different attaching factors, i.e., C-type lectins, T-cell immunoglobulin and mucin domain 1, tyrosine kinase receptor Axl, Niemann-Pick C1			*HLA-DR3* [104] *HLA-A11/DR1* [105] *HLA-A2* [106]	NSG-A2 [107,108] NSG-SGM3 [109,110]
Zika	Glycosaminoglycans NCAM1			*hSTAT2* [111] *IFNAR^−/−^/HLA-B, A, DRB1* [101,112,113]	

## 3. Human Cell Surface Receptor Supplemented Mice

Human pathogenic virus’ species specificity and tissue tropism is often a result of host-specific unique expression patterns of cell surface receptors needed for virus entry. Due to genetic differences of humans and mice, genes encoding necessary cell surface receptors can be lacking or significantly differ in mice. This often results in decreased susceptibility of mice for human pathogenic viruses. This drawback can be overcome by transgenic mice expressing humanized virus entry receptors as a result of genetic modification. 

The current SARS-CoV-2 pandemic is an excellent example of the urgent need of susceptible mouse models for emerging human pathogenic viruses to gain insights into virus biology and to develop antiviral strategies and vaccinations. Earlier studies suggested angiotensin-converting enzyme 2 (ACE2) as essential entry receptor for SARS-CoV viruses [114]. Sequence alignment of human and murine ACE2 revealed changes in five out of eight amino acids relevant for SARS-CoV-2 spike (S) protein binding resulting in poor infection efficiency in mice. Transgenic mice expressing the human *ACE2* gene under a *human cytokeratin 18* (*K18*) promoter in the epithelium of lung, colon, liver, and kidneys [88] were originally generated to study SARS-CoV infections and pathology. These transgenic mice were amongst the first tested as suitable animal model for SARS-CoV-2 infections, resulting in high virus infection levels, viral spread to other organs, decline in pulmonary function and lethality of infected mice. Thus, virus-induced pathology shared many features with human COVID-19 disease [89,90]. Others generated transgenic humanized *ACE2* mice by either microinjection of the *hACE* gene under the murine *ACE2* promoter [91] or by using CRISPR/Cas9 knock-in technology, replacing the endogenous murine *ACE2* with the human *ACE2* [92]. These transgenic mouse models are supposed to exhibit the most natural expression pattern and tissue expression profile of ACE2. 

Recently, studies demonstrated interaction of ACE2 with the transmembrane protease/serine subfamily member 2 (TMPRSS2) which proteolytically cleaves ACE2 and the viral S protein. This interaction seems to be essential for viral infection efficiency [115]. Thus, recent studies suggest approaches to create SARS-CoV-2-sensitive transgenic mice based on *hACE2* and *hTMPRSS2* under the murine *TMPRSS2* promoter in epithelial cells by applying CRISPR/Cas9 technology [115].

Another successful and well-studied example of how to facilitate infection of nonpermissive mice with human pathogenic viruses, is the generation of transgenic mice expressing the human entry receptors for poliovirus (PV) infection. PVs are highly pathogenic for humans and are known to be the causative agent of human paralytic poliomyelitis, which is developed in approximately 1% of infected individuals [116,117]. In humans, PV has a primary tissue tropism for epithelial cells in the pharyngeal and intestinal mucosa. PV can also infiltrate the central nervous system via the blood stream and possesses a distinct cell tropism for motor neurons, which are known to highly express the PV receptor (PVR or CD155) [116,118]. Viral infection necessarily requires surface exposition of human PVR. Thus, already in the early 90s transgenic mice expressing the human *PVR* gene were successfully generated by germline modifications to allow in vivo studies of PV infection and manifestation of poliomyelitis [59,60]. Transgenic mice are permissive for virus infection via parenteral routes and demonstrate neuronal histopathology comparable to paralysis in humans. These studies were of great importance for the analysis of PV neurovirulence and have been used for the investigation of antiviral approaches. However, the virus does not replicate in the alimentary tracts of *hPVR*-tg mice after oral administration, which is the primary route of infection in humans [59,60,61]. When *hPVR* was expressed under the promoter of the murine orthologue *Tage4*, mice were more susceptible to oral PV infection. However, only mice of an age of up to three weeks developed symptoms of poliomyelitis even though these mice were more susceptible to oral infection compared to mice expressing *hPVR* under the human promoter [62]. Several other attempts were made to express PVR in the alimentary tract of mice by expressing *PVR* under the control of diverse promoters [63,64]. Unfortunately, none of these attempts resulted in a transgenic mouse model highly susceptible to oral virus infection. These results suggest that the expression of *hPVR* in the intestine is not solely responsible for infection establishment and indicate that other host factors might be involved in efficient viral infection and tissue permissiveness. 

Interestingly, it was shown that PV is not able to interfere with the murine interferon (IFN) system. Thus, transgenic mice expressing the human *PVR* and lacking the *alpha/beta interferon receptor* (*IFNAR*) gene were generated [119]. These mice were the first to be susceptible to oral infection and exhibited efficient viral infection and replication. This study showed that IFN-induced immune responses play an important role in the infection and multiplication of orally administered PV in the small intestine. However, uncontrolled PV replication also caused severe lesions in tissues that are not susceptible to PV infection in immunocompetent humans, nonhuman primates or mice [65]. This is one out of many examples of how combinations of human transgenes and knockouts in antiviral host genes allows infection of rodents even though tissue tropism and disease outcome are not necessarily reflecting the human disease phenotype. Comparable approaches for human receptor-transgenic mice deficient in IFN-induced immune responses were also applied for studies of other human pathogenic viruses such as measles viruses (Table 1) [69,70]. Nevertheless, these models primarily only allow for productive virus infection of mice but do not fully resemble the true mechanisms underlying host permissiveness and antiviral host processes.

## 4. Transgenic Integration of Human Host/Immune Response Factors

Not only the expression and presence of surface receptors have a decisive influence on the permissiveness of mice for human pathogenic viruses. Differences in host determinants and intracellular antiviral responses also play an important role in the different outcome of virus replication, susceptibility of the organism to certain viruses as well as disease pathology in mice versus humans.

Several human pathogenic viruses such as influenza A viruses (IAV) have the capability to infect laboratory inbred strains of mice, such as BALB/c, C57BL/6 or DBA/2j [120]. Amongst the best-studied mouse-adapted IAV strains are H1N1 A/Puerto Rico/8/34 (PR8) or A/WSN/1933 (WSN). Thus, inbred mice are *per se* permissive for various, though not all, influenza viruses, although mice do not belong to the group of natural hosts of IAV. Within the last decade, mouse models have played a significant role in understanding pathogenesis and molecular mechanisms underlying IAV infection. Nevertheless, it was shown that infection selectivity based on individual patterns of 2,3- and 2,6-linked sialic acid residues, serving as entry receptors for avian and human IAVs respectively, varies in respiratory cell populations in mice and humans [121,122]. 

Additionally, depending on the infectious dose given and the strains used, IAV infection shows a more severe disease phenotype and lethality in mice compared to humans. Studies suggested that the IFN-regulated *Mx* gene plays a major role in innate immune responses to control IAV infection. While the majority of wild type mice express a functional *Mx* gene and, thus, show resistance to IAV infection, several inbred mouse strains demonstrate large deletions or nonsense mutations within the *Mx* locus [123,124]. Compared to normal inbred mice, genetically engineered mice of the same background carrying a functional *Mx* gene survived otherwise lethal doses of 1918 H1N1 pandemic and avian H5N1 IAV [125] (extensively reviewed in [126,127]). These data evidenced the role of antiviral host factors including the intracellular IFN system and IFN-induced genes such as *Mx* in the restriction and regulation of IAV infections. 

However, murine and human antiviral host factors can essentially differ and mice expressing murine *Mx* genes do not necessarily reflect human antiviral intracellular immune responses. These innate restriction factors represent effective species barriers that need to be overcome by viruses to invade the human host. IAV poses a constant threat to humans since zoonotic viral transmissions can cause severe disease and may give rise to pandemics. Thus, transgenic mice expressing the entire human *Mx* locus could be explicitly used to investigate the role of human MxA as a restriction factor that must be overcome by zoonotic IAV strains. Interestingly, these mice exhibited a higher resistance against avian H5 an H7 viruses but were readily susceptible to seasonal human IAVs of H1N1 or H3N2 origin that already efficiently adapted to the human host [93]. 

Not only cell intrinsic immune response factors, but also factors involved in cellular innate and adaptive immune responses are essential for IAV-induced pathology and present a critical component of antiviral immunity. Especially the emergence of zoonotic IAV with surface glycoproteins that are distinct from seasonal human strains pose a risk and demand preclinical systems to investigate vaccination approaches and their efficacy in humans. Human transgenic mice expressing the human MHC class I allele HLA-A*0201 [94,95] or the MHC class II allele *HLA-DR3* [96] provided substantial benefit for screening of different vaccine candidates against IAV [94,95,96]. Furthermore, these transgenic mouse models paved the way for vaccination studies and analyses of immunogenic virus epitopes for various other human pathogenic viruses such as Zika or Ebola (Table 1) [128,129,130]. Despite the great progress that has been achieved with these transgenic mouse models, specific characteristics of immune cells and especially interactions between cells of the innate and adaptive immune responses still largely differ between mice and humans. In particular, activation processes and marker expression of cells of the immune response as well as the efficacy of antiviral approaches can be insufficient or fundamentally different in mice compared to humans.

## 5. Transgenic Immune System Humanized Mouse Models

Humanized mice with functional human cell or tissue engraftment have advanced research in the field of human pathogenic virus infections. Engraftment of human hematopoietic stem cells (HSCs) that develop into functional human immune systems even allows the study of bloodborne virus infections as well as virus-induced immune responses and immunopathology-mediated disease pathogenesis, bridging the gap between rodent models and the clinics.

Currently, there are few major mouse models used with varying degrees of immunodeficiency resulting in different levels of graft acceptance and tolerance for human cells (extensively reviewed in [131,132]). The nonfunctional *protein kinase*, *DNA-activated*, *catalytic polypeptide* (*PRKDC*) gene, resulting in severe combined immunodeficiency (*scid*), or knockout of *recombination-activating genes* (*RAG*) lead to impaired development of T and B cells [133,134,135]. Combination with mutations at the *interleukin-2 receptor common gamma chain* (*IL-2rγ*) allows for even more efficient human cell and tissue engraftment due to severe impairments in multiple cytokine complexes and a profound defect in natural killer (NK) and NKT cell development [136]. Immunodeficiency can be introduced in different genetic backgrounds, however, use of nonobese diabetic (NOD) mice was shown to further increase immune tolerance and even resulted in defective macrophage and dendritic cell (DC) function [137,138,139,140]. Most commonly used immunodeficient mice are: NSG (NOD *scid* gamma), NRG (NOD *Rag* gamma) and NOG (NOD *scid* gamma) mice. The latter contain a *IL2rg* null mutation that does not even allow for cytokine binding. There are three conventional ways to engraft immunodeficient mice with functional human cells: (i) intravenous injection of human bone marrow, cord blood, or granulocyte-colony-stimulating factor (G-CSF) cytokine-mobilized peripheral blood mononuclear cells (PBMCs) which are all rich in human HSC activity, leads to the engraftment of a naive human hemopoietic system in the murine recipient (HSC model) [141,142]. (ii) Engraftment can be achieved by injection of human PBMC, spleen or lymph node cells into immunodeficient mice (PBMC or PBL model) [141,142,143,144]. (iii) To establish BLT (bone marrow, liver, thymus) humanized mice, fetal liver and thymus fragments are implanted under the renal capsule in irradiated adult immunodeficient mice, and HSCs derived from the same fetal liver are injected intravenously [145,146]. 

Aforementioned humanized mouse models with fully functional human immune responses have been applied for studies with different human pathogenic viruses. However, although humanized mice with engrafted human cells provided suitable tools to study human-specific pathogens in the past, these models have been shown to possess some major drawbacks such as development of graft versus host disease, the absence of erythrocytes and neutrophils within reconstituted human immune systems, low numbers and functionally impaired human myeloid cells, dominance of immature B cells as well as minimal production of antigen-specific IgG class antibodies [146,147,148,149]. Many recent advances in this area have addressed these limitations and include the derivation of mouse strains transgenic for human cytokines or HLA alleles, allowing for enhanced human cell engraftment and immune responses [148,150]; extensively reviewed in [132]. Most prominent examples are for instance: huNOG-EXL mice, immunodeficient NOG mice expressing human GM-CSF and human IL-3 cytokines to support myeloid lineage engraftment. These animals show higher overall engraftment levels of human HSCs with higher levels of myeloid cell differentiation [151]. Furthermore, NSG-SGM3 triple transgenic mice express human *IL-3*, *GM-CSF* and *stem cell factor* (*SCF*), therefore combining the features of the highly immunodeficient NSG mouse with the expression of cytokines that support the stable engraftment of myeloid lineages and regulatory T cell populations [152]. Another example for more efficient human cell engraftment is the MISTRG mouse model. These highly immunodeficient mice express the human *signal-regulatory protein alpha* (*SIRPα*), protecting human cells from being phagocytosed. Finally, cytokine-encoding genes *M-CSF*, *IL-3*, *GM-CSF* and *thrombopoietin* are humanized by knock-in replacement, resulting in defects in mouse cell populations dependent on the respective mouse cytokine and, thus, support for engraftment of transplanted human cells [153]. NSG-*HLA-A2*/HHD immunodeficient mice express human HLA class 1 heavy and light chains allowing for the generation of functional human T cell subsets with HLA-restricted immune responses [154]. DRAG mice are NRG animals with chimeric human-mouse class II transgenes encoding the HLA class II antigen binding domain molecules (defined by the *HLA-DR4* genotype) fused to the I-Ed MHC class II molecule. The presence of these *HLA-DR4-IE* transgenes allows enhanced *HLA-DR*-matched HSC engraftment and subsequent human T and B cell development [155]. Interestingly, mice coexpressing HLA-DR4 and HLA-A2 molecules (DRAGA mice) did not differ in their ability to develop human T and B cells, to reconstitute cytokine-secreting CD4+ T and CD8+ T cells, or to undergo immunoglobulin class switching [156]. Most recently, HUMAMICE were developed, the only mouse line combining the knockout of both murine MHC class I and II with subsequent expression of human *HLA-A2* and *HLA-DR1* providing a platform for the generation of humanized models that better resist graft versus host disease [157]. 

These different humanized transgenic mice are currently in use to model the human immune system in scenarios of health and pathology, and serve as a basis for the analysis of human pathogenic viruses, enabling the evaluation of therapeutic candidates in an in vivo setting more relevant to human physiology, in the future.

## 6. Transgenic Humanized Mouse Models for Human-Specific Bloodborne and Hepatotropic Viruses

Improvement of humanized mouse models has opened up a new avenue for studying bloodborne viruses such as HIV as well as hepatitis B and C viruses, where knowledge was previously gained in viral gene/genome transgenic mice. In addition, hemorrhagic fever-inducing viruses such as dengue virus (DENV) or Ebola could be finally investigated in vivo by application of the aforementioned mouse models that allow for human immune system induced pathology. 

The most extensively studied virus in transgenic immune system humanized mice is HIV. HIV research is confronted with various challenges such as the species barrier that was overcome by engraftment of human fetal thymic or lymph node implants allowing for acute infection. However, the RNA genome of HIV is reverse-transcribed into DNA and integrated into the host genome, resulting in latent infections difficult to clear by the host immune system. Additional humanization and especially the use of BLT mice finally allowed for the modelling of HIV transmission via vaginal and rectal routes, latency and a dynamic interplay of HIV-specific cellular immunity and viral escape from immune pressure (reviewed in [158,159]). For instance, HIV-infected MISTRG mice showed lymphocyte activation and changes in lymphocyte subsets in blood and spleen, recapitulating hallmarks of HIV infection in humans [56]. Recently, a study demonstrated that intravaginal HIV infection of DRAG mice, whose mucosal tissues are highly enriched for human lymphocytes expressing the receptors and cofactors needed for HIV entry and replication, resulted in replication competent viruses that spread rapidly. Bone marrow, gut, female reproductive tract and lymph nodes as well as plasma were positively tested for viral RNA. Observation of viral persistence in diverse tissues such as the bone marrow, lymph nodes, and the brain indicated potential sanctuary sites for HIV to escape the host immune system [57]. Furthermore, HIV productively replicated in humanized NOG-EXL mice, and infection induced a decrease in the percentage of CD4+ T cells, inversion of the CD4:CD8 ratio, and changes in cell populations, such as monocytes and dendritic cells, that recapitulated the pathology found in human infection [58].

Preclinical studies on mosquito-borne DENV causing systemic disease have proven to be a difficult task because the virus demonstrates a tropism for different subsets of human cells [160]. In addition, several clinical isolates of DENV do not replicate or cause pathology in nonhuman hosts. Nonhuman primate models were of limited use due to the absence of clinical signs of dengue fever, while mice are naturally resistant against DENV, as the virus is not capable to inhibit murine IFN signaling. Different knockout and transgenic mouse models have been utilized to investigate different DENV research questions. However, due to significant drawbacks, these models did not lead to major breakthroughs in the understanding of human dengue fever. An additional aggravating factor in research on DENV infection is that previous immunity is considered a major risk factor for the development of severe hemorrhagic fever and most severe symptoms are observed at the peak of the human antiviral immune response (reviewed in [161,162,163]). Thus, humanized mouse models have become the most common models used for the study of DENV and the rapid development of new transgenic human–mouse hybrid models recently increased insights into DENV infections. Infection of humanized mice represented some clinical manifestations also observed in humans such as viremia, fever, rash, thrombocytopenia, and erythema. However, since pre-existing immunity is a well-known risk factor for the development of severe dengue disease, it is important that the infection of humanized animal models results in development of humoral and cellular immune responses as observed in humans following primary DENV infection. NSG-A2 mice showed efficient viral replication and IgM antibodies directed against the envelope protein were detected in sera of mice. Furthermore, splenocytes isolated from DENV-immunized mice showed induction of human DENV-specific T cell responses evidenced by IFN γ, IL-2, and TNF-α secretion after stimulation with three known HLA A2-restricted dengue virus-specific CD8 T cell epitopes [102]. When compared with NSG-BLT mice, transgenic NSG-SGM3-BLT demonstrated per se higher levels of mature naive (resting) B cells and lower levels of transitional B cells. Consistently, after DENV infection, higher levels of antigen-specific IgM and IgG were observed, suggesting an enhanced development and maturation of human B cells that can be used for studies in human antigen-specific B cell responses [103]. However, experiments published using humanized mouse models for DENV infection have failed to consistently demonstrate plasma leakage and hemorrhagic phenomenon seen in severe human DENV infection, indicating the need of additional humanization of the vascular system.

In the past years, human-associated viruses have been extensively studied in mice with humanized immune systems. Nevertheless, study of hepatotropic pathogens such as hepatitis B and C viruses remained elusive due to the lack of human hepatocytes in the mouse liver. Human liver chimeric mice are still the gold standard for allowing hepatotropic infections, even though the highly immunocompromised status of these engrafted mice precluded liver pathogenesis mediated by human immune surveillance. Thus, transgenic mice with dual humanization of immune system and liver were established (reviewed in [164,165]). However, the best animal strains for hepatocyte transplantation are not optimal for human HSC engraftment, and vice versa. To promote engraftment of human hepatocytes, transgenic mice expressing a fusion protein of the FK506 binding protein (FKBP) and caspase 8 under control of the albumin promoter (*AFC8*), which induces mouse liver cell death after administration of synthetic drug AP20187, were used. *AFC8*-hu HSC/Hep mice supported HCV infection in the liver and generated a human immune T cell response against HCV antigens. HCV infection induced liver inflammation, hepatitis, and fibrosis, which correlated with activation of stellate cells and expression of human fibrogenic genes [76,77]. Nevertheless, in this mouse model, no significant HCV viremia was detected in the blood, a prerequisite in blood borne human infection.

The A2/NSG-hu HSC/Hep mouse, a humanized mouse model with both human immune system and human liver cells by reconstituting immunodeficient human *HLA-A2* transgenic mice with human hematopoietic stem cells and liver progenitor cells, supported HBV infection and establishment of persistent infection. Human immune responses, albeit impaired in the liver, chronic liver inflammation and liver fibrosis were detected in infected animals. In addition, application of an HBV neutralizing antibody efficiently inhibited HBV infection and associated liver diseases in humanized mice. Furthermore, HBV-mediated liver disease was found to be associated with high levels of infiltrated human macrophages with an M2-like activation phenotype, which has been shown to be associated with accelerated liver fibrosis and necrosis in patients with acute HBV-induced liver failure [85].

Although these transgenic humanized mouse models have not been used extensively in research on human pathogenic viruses yet, their benefit especially in drug and vaccination studies is undeniable. Interestingly, it already has been shown that DRAGA mice can be used to generate cross-reactive, human anti-influenza monoclonal antibodies (hu-mAbs). DRAGA mice were found to be suitable for influenza virus infection, as they can clear a sublethal infection and sustain a lethal infection with influenza virus. A single administration of viral hemagglutinin protein-specific hu-mAbs in IAV-infected DRAGA mice significantly delayed lethality by reducing lung damage [97]. 

## 7. Conclusions and Future Directions

Successful establishment of transgenic and especially transgenic humanized mice provided immeasurable opportunities to advance medical research on infectious diseases that were previously inaccessible. The transgenic mouse models described in this review are, amongst others, groundbreaking for the understanding of human pathogenic virus infections and their disease pathology. Along that line, these mouse models provide the possibility to study emerging virus-induced diseases, as discussed for SARS-CoV-2. Since some viruses have the unique ability to cross species boundaries and adapt to new hosts, humanized mouse models will continue to play a crucial role in understanding newly emerging or human-adapting viruses, in the future. However, existing models are still far from being perfect. A mouse model that can be applied to study all known and newly emerging human pathogenic viruses while completely mimicking virus-induced disease pathogenesis does not exist and researchers still need to consider the scientific questions in the respective areas of the disease to select the best suitable animal model. While virus entry receptor or antiviral host factor humanized mice are a great model to study virus infection, replication and the role of single human genes involved in antiviral immune responses, they are not suitable to evaluate disease pathogenesis or efficacy of antiviral approaches as the complexity of human immune responses is not necessarily reflected. Especially the analysis of possible vaccination candidates or antiviral approaches additionally requires immune system humanized mice. Amongst these, HUMAMICE are highly sophisticated humanized models that allow e.g., to study antigen specificity of human immune cells, however, without additional humanization, these animals are not susceptible for infection with human pathogenic viruses such as SARS-CoV-2.

The ideal future model for most human pathogenic virus infections will be an animal model with a reconstituted human immune system presenting antigens in the human HLA context, constitutively expressing human cytokines necessary for efficient immune cell development. In addition, specific human organs or humanized cell types would be needed to mimic viral transmission routes and disease pathology. Such a model does not exist yet, but with the current advances in technology it might be only a matter of time to have it available to study human specific virus infection complexity and to develop more efficient vaccine and treatment strategies. Furthermore, there still is the sustained need to identify and replace human-specific factors that are absent in mice but are needed for optimal human cell differentiation and function to decrease or even prevent the development of graft versus host disease that still occurs in many of the human immune system engrafted models.

Thus, key improvements in specific areas are still required, however, with careful characterization, the future will yield more appropriate preclinical models, supporting more rapid and relevant translational drug discovery and development.
